# Replacing American snacks with tree nuts increases consumption of key nutrients among US children and adults: results of an NHANES modeling study

**DOI:** 10.1186/s12937-017-0238-5

**Published:** 2017-03-07

**Authors:** Colin D. Rehm, Adam Drewnowski

**Affiliations:** Center for Public Health Nutrition, University of Washington, Seattle, WA 98195-3410 USA

**Keywords:** Nuts, Diet quality, Snacks

## Abstract

**Background:**

Replacing typical American snacks with tree nuts may be an effective way to improve diet quality and compliance with the 2015–2020 Dietary Guidelines for Americans (DGAs).

**Objective:**

To assess and quantify the impact of replacing typical snacks with composite tree nuts or almonds on diet metrics, including empty calories (i.e., added sugars and solid fats), individual fatty acids, macronutrients, nutrients of public health concern, including sodium, fiber and potassium, and summary measures of diet quality.

**Methods:**

Food pattern modeling was implemented in the nationally representative 2009–2012 National Health and Examination Survey (NHANES) in a population of 17,444 children and adults. All between-meal snacks, excluding beverages, were replaced on a per calorie basis with a weighted tree nut composite, reflecting consumption patterns in the population. Model 1 replaced all snacks with tree nuts, while Model 2 exempted whole fruits, non-starchy vegetables, and whole grains (>50% of total grain content). Additional analyses were conducted using almonds only. Outcomes of interest were empty calories (i.e., solid fats and added sugars), saturated and mono- and polyunsaturated fatty acids, fiber, protein, sodium, potassium and magnesium. The Healthy Eating Index-2010, which measures adherence to the 2010 Dietary Guidelines for Americans, was used as a summary measure of diet quality.

**Results:**

Compared to observed diets, modeled food patterns were significantly lower in empty calories (−20.1% and −18.7% in Model 1 and Model 2, respectively), added sugars (−17.8% and −16.9%), solid fats (−21.0% and −19.3%), saturated fat (−6.6% and −7.1%)., and sodium (−12.3% and −11.2%). Modeled patterns were higher in oils (65.3% and 55.2%), monounsaturated (35.4% and 26.9%) and polyunsaturated fats (42.0% and 35.7%), plant omega 3 s (53.1% and 44.7%), dietary fiber (11.1% and 14.8%), and magnesium (29.9% and 27.0%), and were modestly higher in potassium (1.5% and 2.9%). HEI-2010 scores were significantly higher in Model 1 (67.8) and in Model 2 (69.7) compared to observed diets (58.5). Replacing snacks with almonds only produced similar results; the decrease in sodium was more modest and no increase in plant omega-3 fats was observed.

**Conclusion:**

Replacing between-meal snacks with tree nuts or almonds led to more nutrient-rich diets that were lower in empty calories and sodium and had more favorable fatty acid profiles. Food pattern modeling using NHANES data can be used to assess the likely nutritional impact of dietary guidance.

## Introduction

Between-meal snacks represent an important source of solid fats, added sugars, and sodium in the American diet [1–6]. The USDA has identified solid fats and added sugars (SoFAs) as the main sources of “empty” calories, contributing ample energy but few nutrients [7, 8]. Snacking between meals is consistently listed among potential causes of obesity and weight gain [9–11] and is an issue of public health concern.

Healthier snacking patterns could improve diet quality by reducing empty calories and sodium, and increasing intakes of some insufficiently consumed nutrients [12, 13], including fiber and potassium [14]. Healthier snack patterns may also shift the fatty acid ratio away from solid fats towards oils, by increasing mono- and polyunsaturated fats (MUFAs and PUFAs), and plant-based omega-3 fatty acids, and reducing saturated and *trans* fat consumption [15, 16]. In the United States, consumption of added empty calories (e.g., added sugars and solid fats) and sodium is much higher than recommended, while consumption of some food groups, including whole grains, plant and seafood protein, whole fruits and vegetables lags well below recommended levels [17–19].

Tree nuts, including almonds, walnuts, and cashews, have been featured prominently in multiple dietary recommendations and guidelines [14], as well as consumer-facing recommendations regarding healthy snacking from numerous profesional and advocacy groups, including the American Heart Association, the American Diabetes Association and Academy of Nutrition and Dietetics [20–22]. In the nationally representative cross-sectional National Health and Nutrition Examination Surveys (NHANES), tree nut consumption was associated with better nutrient adequacy, higher diet quality, and improved health risk markers [15, 16, 23]. Prospective cohorts have also observed that consumption of tree nuts reduces the risk of all-cause mortality, deaths due to heart disease, cancer and respiratory disease, the incidence of heart disease, and may reduce the likelihood of weight gain [24–28]. Analyses of dietary trends suggest that the consumption of nuts/seeds among US adults has risen from 0.43 to 0.69 oz-equivalents per day from 1999–2000 to 2011–2012. In 2011–2012, just over 30% of adults consumed ≥5 servings of nuts/seeds per week, the recommended amount per 2000 kcal/d from the 2015–2020 Dietary Guidelines for Americans [14]. While no specific recommendation exists for tree nuts, the trend in their consumption has been particularly strong, increasing 145% from 0.11 to 0.27 oz-equivalents per day from 1999–2000 to 2011–2012 [17]. Among the reasons for the substantial growth in tree nut consumption may be their well-documented health benefits as well as the certification of a qualified health claim for nuts and heart disease in 2003 [29].

Following earlier studies on food pattern modeling [30, 31], we sought to quantify the nutritional impact of replacing between-meal snacks with tree nuts or almonds. First, a tree nut composite was created based on the relative frequency of consumption within the population (e.g., a weighted average of almonds, walnuts, pecans and other tree nuts). In substitution models, between-meal snacks (excluding beverages) were replaced, on a per calorie basis, with this weighted tree nut composite. One model replaced all solid snack foods and a second model replaced all solid snacks, with the exception of whole fruits, non-starchy vegetables and whole grain foods. Based on the well-documented high nutrient density of tree nuts [32], we expected to observe measurable improvements in the quality of food patterns generated. The primary goal was to quantify the impact of such a replacement on key dietary constituents of public health interest, including added sugars, solid fats, empty calories, fatty acids, sodium, potassium and magnesium.

## Methods

### Study population & dietary data

Dietary data were drawn from two cycles of the nationally representative National Health and Nutrition Examination Survey (NHANES) for 2009–2010 and 2011–2012. Data were available for 17,444 children, adolescents and adults age ≥ 1y. Any children who consumed breast milk were excluded. The sample included 6,881 children and adolescents (age 1–19y) and 10,563 adults (age ≥ 20y). At the time of study initiation (late Summer 2015), these were the latest NHANES dietary data available.

Data from two non-consecutive 24-h recalls were used. The first 24-h recall was completed in-person at the Mobile Examination Center with a trained interview. The second recall was completed over the telephone many days later. The 24-h recall queries all foods/beverages consumed by participants from midnight-to-midnight on the previous day [33]. Eighty-five percent of respondents completed two dietary recalls and the remaining 15% completed a single dietary recall.

Nutrient data was obtained from the Food and Nutrient Database for Dietary Studies (FNDDS); FNDDS 5.0 for 2009–2010 data and FNDDS 2011–2012 for 2011–2012 data were used [34]. Data on consumption of food groups of interest (e.g., nuts/seeds), added sugars, solid fats, and oils were obtained from the Food Patterns Equivalent Database (FPED); the cycle-specific version of FPED was used for each NHANES cycle (e.g., FPED 2009–2010 for 2009–2010 data) [35].

### Modeling strategy

Two models were developed. Model 1 replaced all between-meal solid snacks with tree nuts on a calorie-per-calorie basis. Model 2 exempted between meal snacks consisting of whole fruit, non-starchy vegetables, or grain products containing more than 50% whole grains. The models were applied to solid snacks only and did not replace between-meal beverages, whether fruit juices, soft drinks, cola, or alcohol. Between-meal beverages were not included in the substitution models for two reasons. First, we wanted the substitution models to be a like-for-like comparison, where solid foods were replaced with other solid foods. Second, we wanted to keep the amount of energy being replaced to levels that could feasibly be replaced with tree nuts, by including beverages in the substitution models the amount of energy being replaced would lead to infeasible intakes of tree nuts. Beverage additions (i.e., sugar added to coffee) were not replaced. Diets of individuals already consuming tree nuts as exclusive snacks were not modified. Foods were replaced on a calorie-per-calorie basis to observe the impact on diet quality within an isocaloric context.

**Fig. 1:**
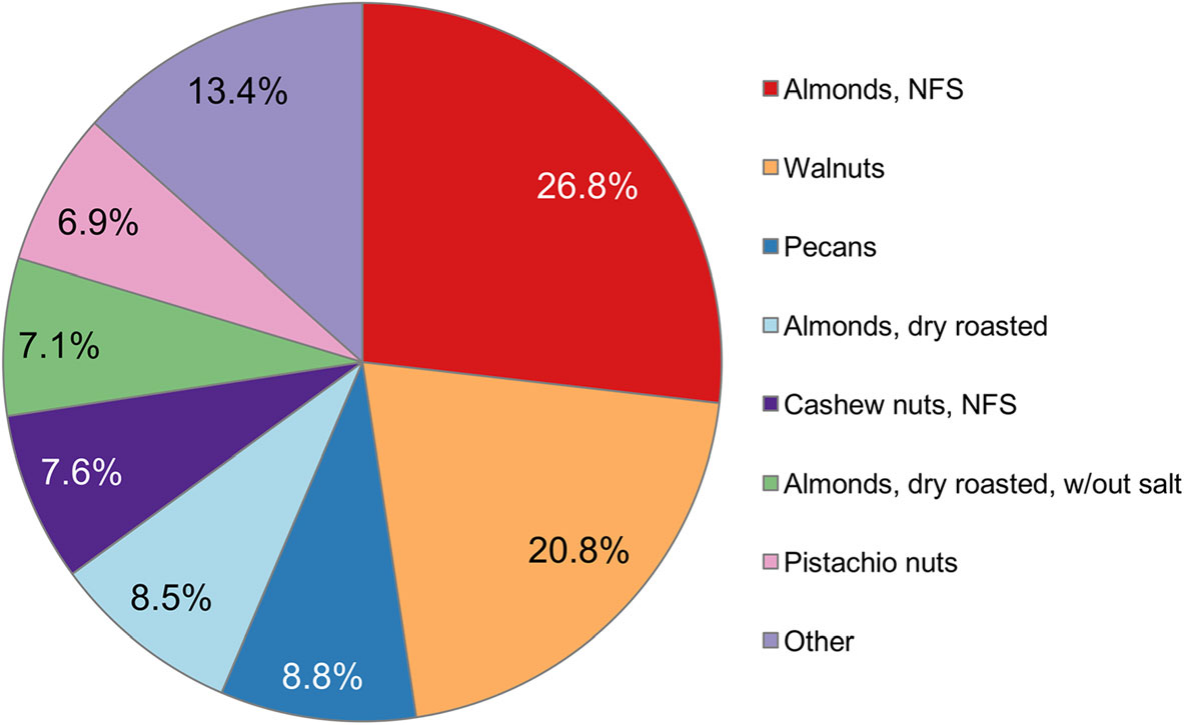
Breakdown of composite tree nut. Value indicates the relative weight of each tree nut type. For example, “almonds, not formally specified” contributed 26.8% to the composite tree nut. Graph shows all tree nuts contributing more than 5%. Other nuts contributing between 1-5% include, cashews (dry roasted), almonds (unroasted), cashews (dry roasted, no salt), and almonds roasted

A composite tree nut was created based on current consumption patterns. First, a list of all tree nuts consumed by NHANES participants from 2009 to 2012 was made (n = 21). Second, the weighted frequency of consumption was estimated and a weight was assigned to each tree nut corresponding to its frequency of consumption. For example, “almonds, not formally specified” were identified as the most frequently consumed tree nut and contributed a weight of 0.268 to the composite tree nut. Other important components of the composite tree nut were walnuts (weight = 0.208), pecans (0.088), dry roasted almonds (0.086), cashews (0.076), dry roasted almonds without salt (0.071), and pistachio nuts (0.069) (see Fig. 1). The nutrient profile for the composite tree nut (per 100 kcal) was then estimated based on these weights. The nutrient profile of the composite tree nut was then used in the models based on the amount of snack energy eligible for substitution. Because almonds were the most frequently consumed tree nut (~44% of all tree nuts), secondary analyses were conducted with a composite almond alone.

The primary outcome measures were dietary outcomes based on nutrients of interest, selected based on their overall importance to current dietary recommendations [14, 36]. Nutrients of interest included dietary fiber, potassium and magnesium; each of which are underconsumed per the 2015–2020 Dietary Guidelines for Americans and the first two are nutrients of public health concern [14]. Furthermore, the 2010 Healthy Eating Index (HEI-2010) was examined as a summary measure of diet quality to measure the global impact of the models on diet quality. The HEI-2010 is an energy-adjusted measure of diet quality based on 12 components, including 9 components to encourage, including total fruit, whole fruit, total vegetables, greens and beans, whole grains, dairy, total protein, protein from plant and seafood sources and the fatty acid ratio (favoring higher ratio of mono- and polyunsaturated fatty acids to saturated fatty acids); and 3 components to discourage, including refined grains, energy from solid fat and added sugars, and sodium. Details on the HEI-2010 algorithm have been previously described [37]. A version of the Healthy Eating Index corresponding to the 2015–2020 Dietary Guidelines for Americans is not yet available, but the 2010 and 2015–2020 Dietary Guidelines are generally in alignment regarding nutrients/food groups whose consumption should be encouraged and/or limited [14, 38].

### Analyses

The National Cancer Institute (NCI) method was used to characterize the usual intake of nutrients and food groups of interest as observed and for each of the two substitution models [39, 40]. This method can be used to estimate the usual intake of nutrients and food groups, including the population-distribution of intakes. Two models were fit using this method; one for ubiquitously consumed nutrients (i.e., foods/nutrients consumed by most individuals on all days, such as fiber) and another, which incorporates both the mean and probability of consumption for episodically consumed foods/nutrients (i.e., not consumed by most individuals on all days, such as whole grains). Additional covariates were included in the model to account for whether the recall data were from a weekday or weekend and whether it was the first or second recall, which accounted for mode (e.g., telephone vs. in-person) and order effects. Estimates of the population mean and standard error and distribution of intakes were conducted for observed diets and each modeling scenario for the entire population and by age group. The NCI method makes uses of both the first and second recall, and includes all individuals regardless of whether they completed a second recall. To estimate population means for the HEI-2010, the population ratio method was used, which uses a single recall [37, 41]. Code developed by the United States Department of Agriculture was used to estimate the population average HEI-2010 and HEI-2010 component values for the observed diet and two models [42].

In order to account for the complex survey design of NHANES data, balanced repeated replication (BRR) weights were constructed using WesVar software (Westat, Rockville, MD, 2012) and a Fay’s adjustment of 0.7. A total of 32 BRR runs were repeated for each analysis, making the results representative of the US population. To determine if mean intakes differed for the different models as compared to the observed we conducted survey-weighted t-tests with an unequal variance, comparing the results of each model to baseline intakes. To place the results in context, we defined a relative change of less than 5% to be “marginal” if the difference was statistically significant (*p* < 0.05). Statistically significant differences (*p* < 0.05), for which the relative change was between 5% and less than 10% (or between −5% and greater than −10%), are described as “modest” or “moderate” changes, while statistically significant changes greater than 10% (or less than −10%), are described as “strong” or “dramatic”. Ten percent was selected as the cut-off for “strong” effects as it corresponds to the definition of a “good source” of nutrients or minerals according to the US Food and Drug Administration [43]. A reference line corresponding to the Daily Value (DV) for nutrients with a daily value in units consistent with our analysis are included in all graphics [44]. All output for this paper was generated using SAS software, Version 9.3 and are representative of the US population (SAS Institute Inc. Cary, NC, USA).

### Data availability and ethical approval

The necessary IRB approval for NHANES had been obtained by the National Center for Health Statistics (NCHS) [45]. For adult participants, written informed consent was obtained directly from the participating adult. For child participants, parental/guardian written informed consent was obtained and children/adolescents ≥ 12y provided additional written consent. All data used here are publicly available on the NCHS and USDA websites [46]. Publicly available data, such as those used here per University of Washington policies, do not involve “human subjects” and their use requires neither IRB review nor an exempt determination. According to University of Washington policies, these data may be used without any involvement of the Human Subjects Division or the University of Washington Institutional Review Board.

## Results

Among the 17,444 study participants, 76.9% consumed a snack on their first recall day (defined as any energy from any food or beverage identified as a snack). Overall, mean energy from all snacks was 303 kcal/d, whereas the median was 204 kcal/d. Among snack-consumers, mean energy from snacks was 394 kcal/d, whereas the median was 293 kcal/d. Mean energy from solid snacks eligible for substitution was 252 kcal/d and the median was 148 kcal/d. For Model 1, the amount of solid snack energy in kcal/d that was eligible for substitution depended on age: 250 (ages 1–3y), 341(4–8y), 374 (9–13y), 350 (14–19y), 316 (20–30y), 309 (31–50y), 285 (51–70y), and 206 (71 + y). Median energy from snacks (293 kcal/d) corresponds to 51 g of almonds or 45 g of walnuts.

The distribution of calories from solid food snacks is presented in Table 1 for snacks eligible for replacement under Model 1 and Model 2, respectively. Under both substitution scenarios, cookies and brownies, ice cream and frozen dairy desserts, cakes and pies and candy containing chocolate were the primary sources of snack calories from solid food. Popcorn, apples, bananas and other fruits and fruit salads each contributed more than 1% of snack calories from solid foods.

**Table 1:** Summary of snack calories by food category, NHANES 2009-2012

	% of solid food snack calories from each food group under Modeling scenarios	
Food Category^a^	Model 1^b^	Model 2^b^
Cookies and brownies	11.2%	13.5%
Ice cream and frozen dairy desserts	9.1%	10.9%
Cakes and pies	7.2%	8.6%
Candy containing chocolate	5.9%	7.1%
Tortilla, corn, other chips	4.9%	4.8%
Candy not containing chocolate	4.1%	4.9%
Crackers, excludes saltines	3.9%	4.2%
Potato chips	3.6%	4.3%
Doughnuts, sweet rolls, pastries	3.6%	4.3%
Popcorn	3.5%	-
Cheese	3.3%	4.0%
Yeast breads	2.6%	2.6%
Apples	2.0%	-
Pretzels/snack mix	1.9%	2.3%
Bananas	1.8%	-
Pizza	1.7%	2.0%
Ready-to-eat cereal, higher sugar (>21.2 g/100 g)	1.5%	0.8%
Cereal bars	1.4%	0.4%
Yogurt, lowfat and nonfat	1.3%	1.6%
Biscuits, muffins, quick breads	1.3%	1.5%
Cold cuts and cured meats	1.1%	1.3%
Other fruits and fruit salads	1.1%	-
Burritos and tacos	0.9%	1.1%
Other foods	21.0%	19.7%

^a^Food categories from USDA What We Eat in America Food Groups

^b^Snacks eligible for replacement in Model 1 include all solid foods; snacks eligible for replacement in Model 2 include all solid foods, with the exception of non-starchy vegetables, whole fruit, and foods where more than 50% of the grain content comes from whole grains. The USDA considers popcorn to be a whole grain

### Results of primary models

**Fig. 2:**
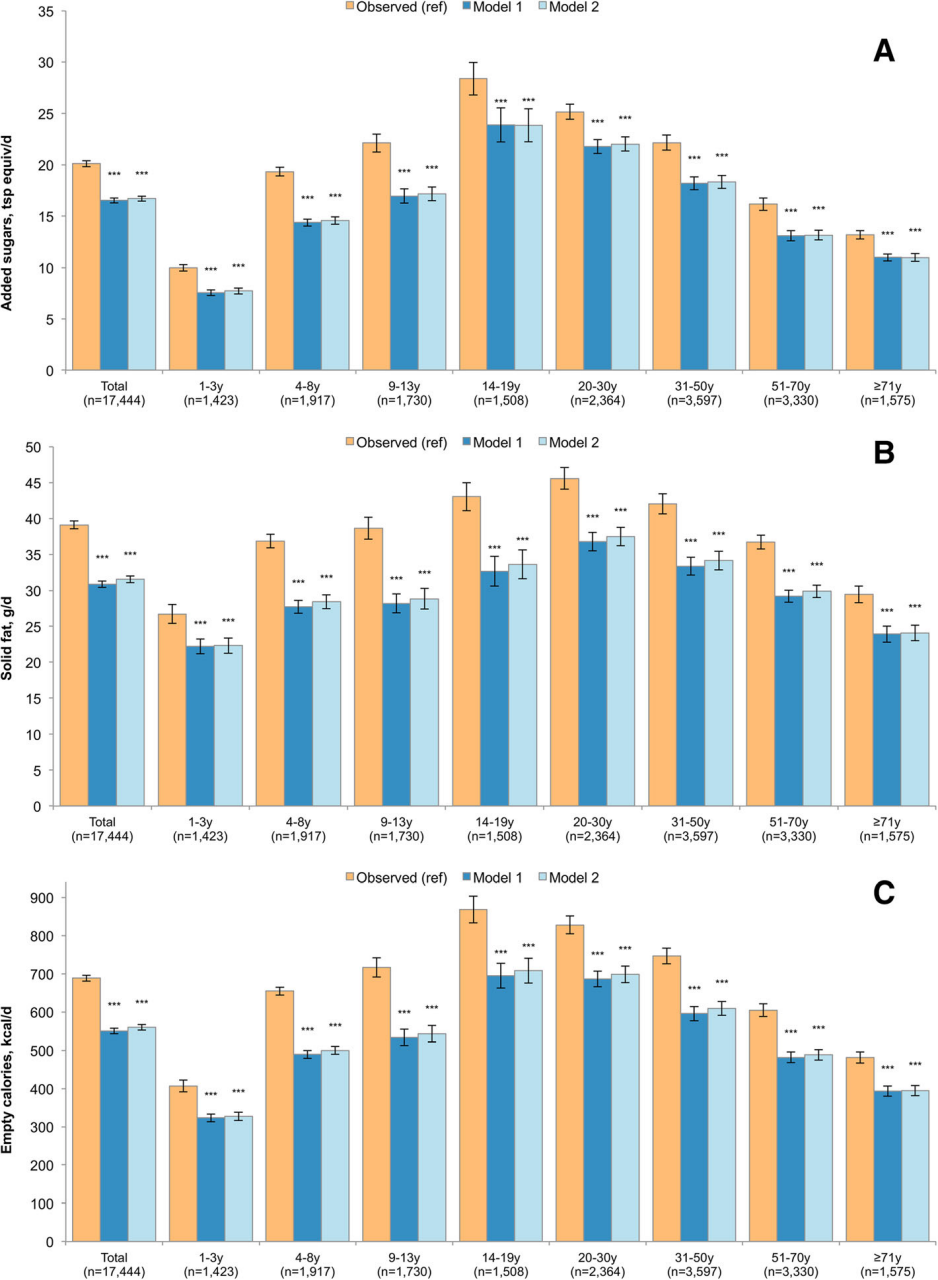
Added sugars (Panel **a**), solid fats (Panel **b**) and energy from empty calories (Panel **c**) in observed and modeled diets, overall and by age group. Error bars are 95% confidence intervals. Model 1 is substitution of all solid snack foods with the composite tree nut and Model 2 is substitution of all solid foods except for whole fruit, non-starchy vegetables and foods where more than 50% of the total grain comes from whole grains. *P*-value of difference comparing each model to observed value is indicated by asterisk (***p < 0.001; **0.001 < *p*-value < 0.01; *0.05 < *p* < 0.01). Hypothesis testing comparing Model 1 to Model 2 was not conducted

**Fig. 3:**
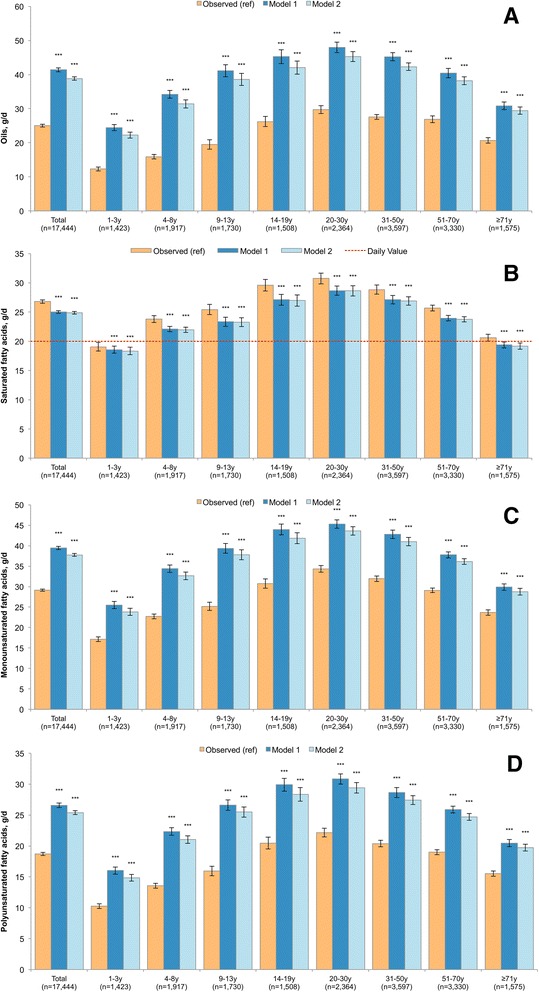
Oils (Panel **a**), saturated fatty acids (Panel **b**), monounsaturated fatty acids (Panel **c**), and polyunsaturated fatty acids (Panel **d**) in observed and modeled diets, overall and by age group. Error bars are 95% confidence intervals. Model 1 is substitution of all solid snack foods with the composite tree nut and Model 2 is substitution of all solid foods except for whole fruit, non-starchy vegetables and foods where more than 50% of the total grain comes from whole grains. *P*-value of difference comparing each model to observed value is indicated by asterisk (***p < 0.001; **0.001 < *p*-value < 0.01; *0.05 < *p* < 0.01). Hypothesis testing comparing Model 1 to Model 2 was not conducted. A reference line is provided for saturated fatty acids, which corresponds to the Daily Value, commonly used on food labels. The Daily Value may not apply to children less than 4y and for pregnant or lactating women

**Fig. 4:**
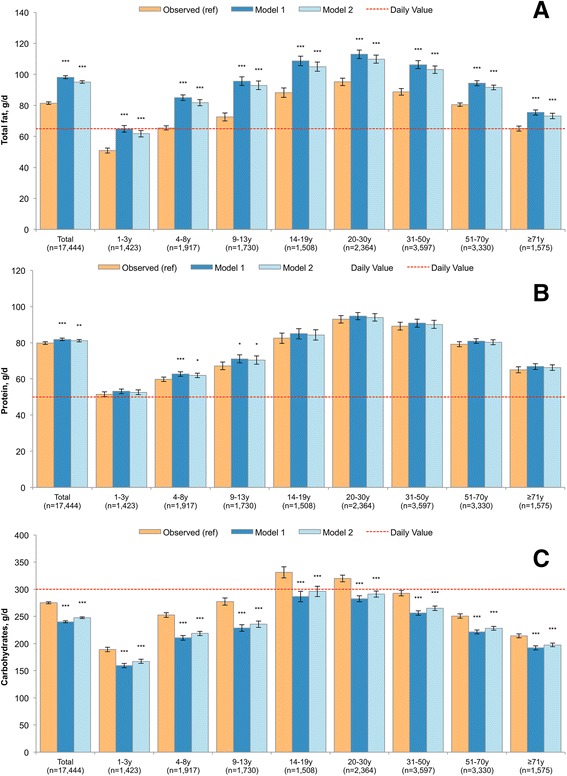
Total fat (Panel **a**), protein (Panel **b**), total carbohydrates (Panel **c**) in modeled and observed diets, overall and by age group. Error bars are 95% confidence intervals. Model 1 is substitution of all solid snack foods with the composite tree nut and Model 2 is substitution of all solid foods except for whole fruit, non-starchy vegetables and foods where more than 50% of the total grain comes from whole grains. *P*-value of difference comparing each model to observed value is indicated by asterisk (***p < 0.001; **0.001 < *p*-value < 0.01; *0.05 < *p* < 0.01). Hypothesis testing comparing Model 1 to Model 2 was not conducted. A reference line is provided which corresponds to the Daily Value, commonly used on food labels. The Daily Value may not apply to children less than 4y and for pregnant or lactating women

**Fig. 5:**
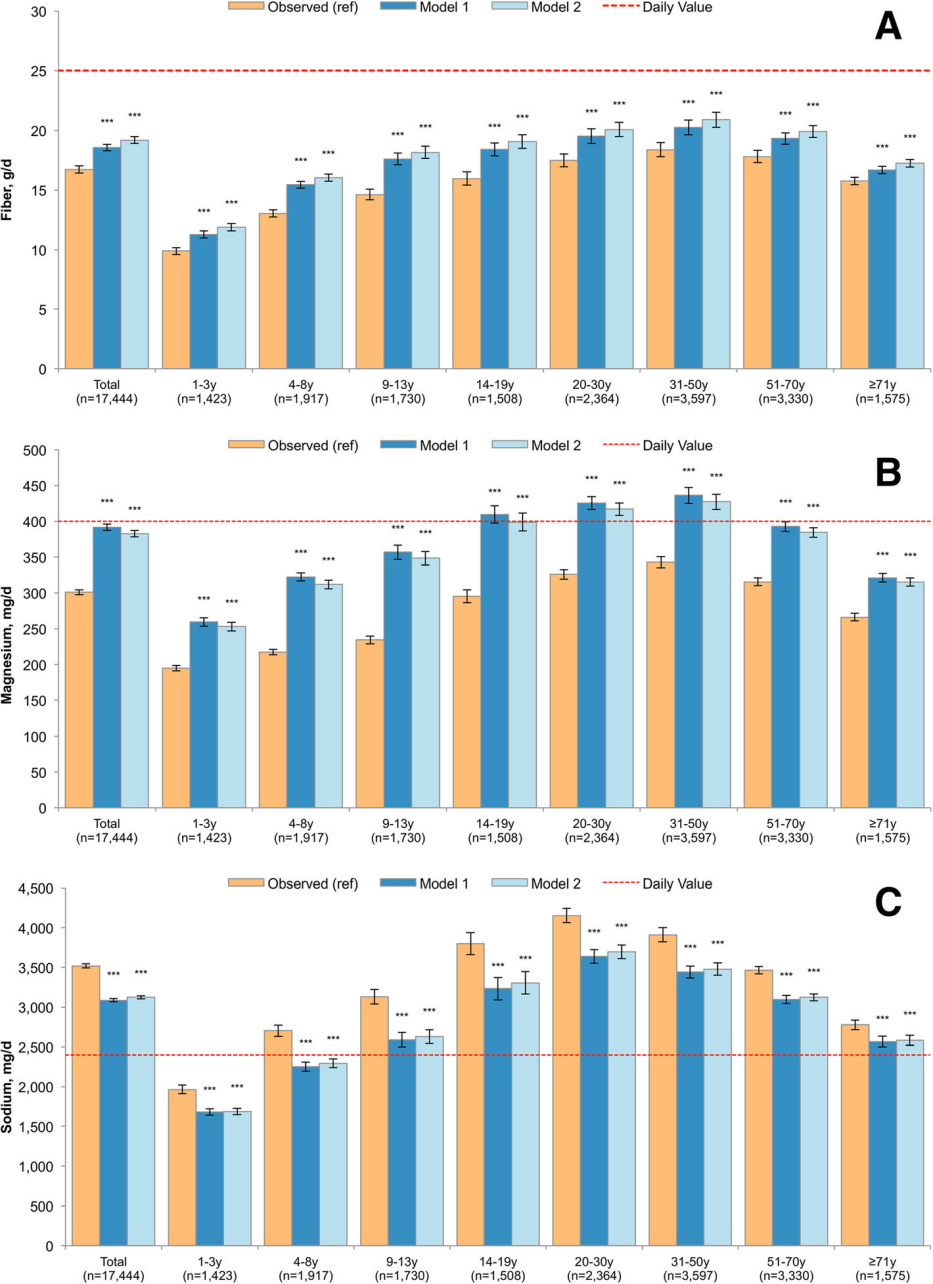
Dietary fiber (Panel **a**), magnesium (Panel **b**), and sodium (Panel **c**) in modeled and observed diets, overall and by age group. Error bars are 95% confidence intervals. Model 1 is substitution of all solid snack foods with the composite tree nut and Model 2 is substitution of all solid foods except for whole fruit, non-starchy vegetables and foods where more than 50% of the total grain comes from whole grains. *P*-value of difference comparing each model to observed value is indicated by asterisk (***p < 0.001; **0.001 < *p*-value < 0.01; *0.05 < *p* < 0.01). Hypothesis testing comparing Model 1 to Model 2 was not conducted. A reference line is provided which corresponds to the Daily Value, commonly used on food labels. The Daily Value may not apply to children less than 4y and for pregnant or lactating women

The nutrient content of modeled food patterns is presented in Figs. 2, 3, 4 and 5 for the entire population and by age group. Model 1 and Model 2 showed significant declines in consumption of empty calories (−20.1% and −18.7%), and a significant drop in solid fats (−21.0% and −19.3%) and in added sugars (−17.8% and −16.9%). Consistent with the decline in solid fats, consumption of saturated fatty acids (SFA) declined modestly in both models (−6.6% and −7.1%). By contrast, the consumption of oils (+65.3% and +55.2%), total polyunsaturated fatty acid (PUFA, +42.0% and +35.7%), alpha-linolenic acid (ALA, +53.1% and +44.7%) and monounsaturated fatty acid (MUFA, 35.4% and 29.6%) all increased. The significant increase in total fat (+20.5% and +16.8%) was accompanied by a greatly improved mono- and polyunsaturated to saturated fat ratio. Dietary carbohydrates dropped significantly (−13% and −10%), whereas a small but statistically significant increase was observed for protein (+2.6% and +1.7%).

Sodium declined from 3,518 mg/d in observed diets to 3,086 mg/d (−12.3%) and 3,124 mg/d (−11.2%) in Model 1 and 2, respectively. For dietary fiber, an 11.1% and 14.8% increase was observed compared to observed diets. Small changes were observed for potassium (not shown, +1.5% and +2.9%) and strong increases were observed for magnesium (+29.9% and +27.0%).

### Change in percent of population meeting recommendations

A change in the proportion of individuals meeting pre-specified dietary recommendations is an alternative way of assessing diet quality. For sodium, the percent of the population consuming less than 2300 mg/d nearly doubled from 11.7% as observed to 21.6% and 20.4% in Models 1 and 2, respectively. For dietary fiber, the percent of the population consuming more than 25 g/d increased from 10.7 to 15.9% and 18.8% in Models 1 and 2, respectively.

### Age-specific results

Figures 2, 3, 4 and 5 also show the nutrient quality of modeled food patterns for specific age groups. Age-specific effects were impacted by a number of factors, including the frequency of snacking within that age group and the types of snacks typically consumed within that age group. For example, children 4–8y and 9–13y were most likely to consume candy/confectionary as snacks, so their models had a more dramatic impact in reducing sugars. By contrast, older adults were least likely to consume between meal snacks, so the results of snack substitution tended to be weaker in this population as compared to younger adults. Significant declines in empty calories, solid fats and added sugars were observed for all groups, but were most profound among children 4–8y and 9–13y.

### Results of almond-only modeling

Sensitivity analyses evaluated the impact of a snack substitution model using almonds only. Almonds were the most frequently consumed type of tree nut during the NHANES 2009–2012 data collection period (see Fig. 1). Substitution modeling using the tree nut composite and almonds-alone produced very similar results (see Table 2). Significant declines in empty calories, added sugars, and solid fats were observed for modeled food patters in both models. A drop in sodium in the almond-only model was weaker but statistically significant. The only difference was a decrease in dietary alpha-linolenic acid (plant based omega 3 essential fatty acid) in the almond-only model as compared to the composite model. This is likely due to the fact that walnuts contain high levels of alpha-linolenic acid whereas almonds do not.

**Table 2:** Results of almond-only substitution models, NHANES 2009-2012

	Population mean (95% CI)		
	Observed	Model 1^a^	Model 2^b^
Macronutrients			
Total fat (g/d)	81.5 (80.7, 82.2)	97.2 (96.3, 98.1)***	94.1 (93.2, 95)***
Saturated fat (g/d)	26.8 (26.5, 27.1)	24.2 (24, 24.5)***	24.2 (24, 24.5)***
Monounsaturated fat (g/d)	29.1 (28.9, 29.4)	42.9 (42.4, 43.4)***	40.6 (40.2, 41.1)***
Polyunsaturated fat (g/d)	18.7 (18.4, 19)	22.9 (22.6, 23.2)***	22.2 (21.9, 22.5)***
Alpha-linolenic acid (mg/d)	1651 (1626, 1675)	1420 (1399, 1441)***	1442 (1420, 1463)***
Protein (g/d)	79.8 (79.1, 80.5)	83.8 (83.0, 84.5)***	82.9 (82.2, 83.5)***
Oils (g/d)	25.0 (24.6, 25.5)	40 (39.5, 40.6)***	37.6 (37.1, 38.1)***
Solid fats (g/d)	39.1 (38.6, 39.6)	31 (30.5, 31.4)***	31.6 (31.1, 32.1)***
Carbohydrates (g/d)	275 (273, 277)	240 (239, 242)***	248 (246, 250)***
Added sugars (tsp/d)	20.1 (19.8, 20.4)	16.6 (16.4, 16.9)***	16.8 (16.5, 17)***
Empty calories (kcal/d)	689 (681, 696)	553 (546, 560)***	563 (556, 570)***
Vitamins & Minerals			
Fiber (g/d)	16.7 (16.4, 17)	19.9 (19.6, 20.2)***	20.4 (20.1, 20.7)***
Magnesium (mg/d)	301 (298, 305)	424 (419, 429)***	410 (405, 415)***
Potassium (mg/d)	2683 (2655, 2710)	2777 (2751, 2803)***	2814 (2786, 2841)***
Sodium (mg/d)	3518 (3493, 3543)	3220 (3200, 3240)***	3410 (3387, 3433)***

***Indicates that population mean values between the Model and Observed diets were highly statistically significant (*p* < 0.001)

^a^Model 1 replaced all snack foods

^b^Model 2 replaced all snack foods with the exception of non-starchy vegetables, fruits and foods where more than 50% of the grain comes from whole grains

### Impact of models on healthy eating index-2010

**Fig. 6:**
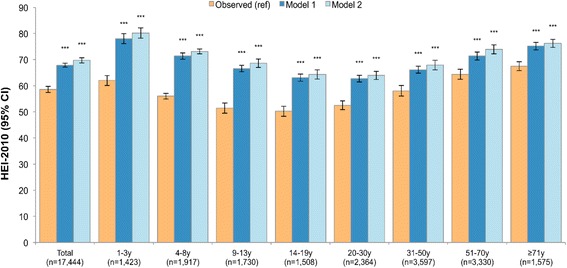
Healthy Eating Index-2010 in modeled and observed diets, overall and by age group. Error bars are 95% confidence intervals. Model 1 is substitution of all solid snack foods with the composite tree nut and Model 2 is substitution of all solid foods except for whole fruit, non-starchy vegetables and foods where more than 50% of the total grain comes from whole grains. *P*-value of difference comparing each model to observed value is indicated by asterisk (***p < 0.001; **0.001 < *p*-value < 0.01; *0.05 < *p* < 0.01). Hypothesis testing comparing Model 1 to Model 2 was not conducted

**Fig. 7:**
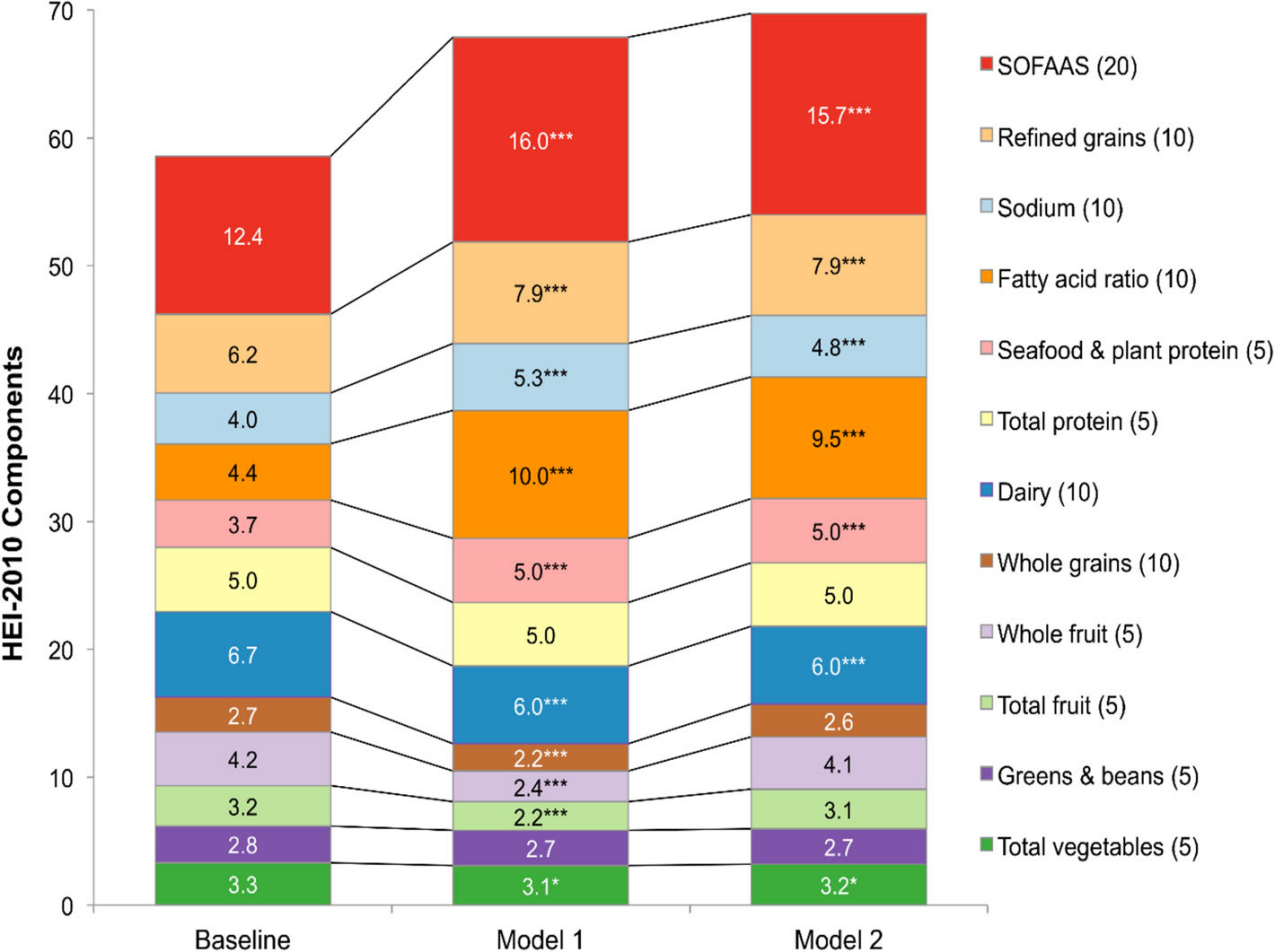
Healthy Eating Index-2010 components in modeled and observed diets. Model 1 is substitution of all solid snack foods with the composite tree nut and Model 2 is substitution of all solid foods except for whole fruit, non-starchy vegetables and foods where more than 50% of the total grain comes from whole grains. *P*-value of difference comparing each model to observed value is indicated by asterisk (***p < 0.001; **0.001 < *p*-value < 0.01; *0.05 < *p* < 0.01). Hypothesis testing comparing Model 1 to Model 2 was not conducted

To measure the total impact of the substitution models on diet quality, the HEI-2010 was examined (see Fig. 6). Based on observed diets, the population mean HEI-2010 value was 58.5 (95% CI 57.4, 59.7). For both models, the HEI-2010 values were significantly higher, 67.8 for Model 1 and 69.7 for Model 2 (*p* < 0.001 for each). For all age groups, the HEI-2010 score was significantly higher in both Model 1 and Model 2 as compared to observed diets. The effect was particularly profound among children and adolescents due to low HEI-2010 baseline values and higher frequency of consuming lower quality snacks. As expected, the results of Model 2 tended to be stronger than for Model 1. When evaluating individual HEI-2010 components (see Fig. 7), modest but statistically significant declines were observed for total vegetables, total fruit, whole fruit, whole grains and dairy comparing observed diets to Model 1. However, component values for seafood and plant protein, fatty acid ratio, sodium, refined grains, and energy from solid fat and added sugars all significantly improved. Similar results by component were observed for Model 2, but with no change in whole fruit, total fruit and whole grains, and modest declines in dairy and total vegetables.

## Discussion

Faced with the need to improve US diets, the 2015–2020 Dietary Guidelines for Americans stressed that making small shifts in eating patterns can make a big difference in diet quality overall [14]. One recommendation was to improve the nutrient quality of between-meal snacks, replacing high-calorie snacks with more nutrient-rich options [14]. In a parallel effort to improve diets of children, new federal rules require healthier snacks in schools [47]. Quantifying the impact of replacing typically consumed solid food snacks with tree nuts was the primary purpose of the present study. For the same amount of calories the modeled food patterns replacing currently consumed solid food snacks with tree nuts were more nutrient dense. First, there was a significant reduction in empty calories. Second, there was a shift in dietary fat profiles. The modeled diets had much less saturated fat and a more favorable fatty acid profile, characterized by more oils, more MUFAs and PUFAs, and more plant omega-3 s (alpha-linolenic acid). The ratio of PUFA and MUFA to saturated fatty acids was much more favorable than in observed diets. Total fat content was higher, as might be expected after replacing many refined grains or sugar-sweetened grains with nuts containing fat, fiber, and protein. Overall, Healthy Eating Index-2010 scores improved in the modeled compared to observed diets.

Frequent between-meal snacking has long been an issue of public health concern [48, 49]. In several past studies, increased snacking frequency has been linked to rising obesity rates [9, 10]. First, more frequent snacking has been associated with consuming more calories overall [8]. Second, some of the commonly eaten snacks were of low, if not minimal, nutritional value [8]. Foods and beverages consumed as snacks provided higher proportions of alcohol, carbohydrates, and total sugars relative to calories, while providing lower proportions of many other nutrients. On average, US adults consumed 24% of daily calories as snacks [49]. Consistent with prior reports [3, 50, 51], the most commonly eaten snacks in the US were sweet desserts high in carbohydrates, sugars, and saturated fats. The top items in the 2009–2012 NHANES were cookies, brownies, cakes and pies, ice cream and frozen dairy desserts, chocolate candy and doughnuts, pastries and sweet rolls. Also on the list were corn chips, crackers and potato chips. Between-meal snacks consisting of apples, bananas, yogurts and other fruit were eaten less often.

Substitution modeling of dietary patterns offers a compelling way to test the nutritional impact of dietary guidance [30]. When based on nationally representative dietary data, such models can provide insight into the impact of federal dietary guidelines on the diet of the entire population or specific population sub-groups [30, 31]. The modeled food patterns can be used to evaluate, compare, and rank the impact of following dietary advice on measures of diet quality at the population level. Results of modeling studies can be used to inform effective nutrition communications and the sub-populations that may benefit most from such efforts. For example, we observed the greatest impact of replacing snacks with tree nuts among children and adolescents, as they tended to consume proportionally more nutrient sparse and less healthful snacks as compared to adults. In addition, children/adolescents are less likely to consume nuts/seeds both overall and as a snack [14].

With that said, such models also have limitations. First, for any substitution model, the units of the substitution model are critical (e.g., whether foods are replaced on per-calorie or per-serving). Given the diverse number of foods in the present study and the arbitrary nature of serving sizes, we opted for an isocaloric substitution model. In simple terms, such a model would result in no change in energy intakes, and therefore no change in body weight. However, if applied in practice, given the relatively high satiety of nuts compared to commonly consumed solid snacks, we might expect fewer calories to be consumed [52, 53]. However, this assumption is not verifiable, so relying on an isocaloric model seems most appropriate. Beyond impacts on weight, substantial improvements to the diet independent of energy changes is likely to have considerable population health benefits. Second, as evidenced by the impact of the models on Healthy Eating Index-2010 component scores, replacement of all solid snacks with tree nuts resulted in modest declines in whole fruit, vegetable, whole grain and dairy intakes. Model 1 was agnostic as to the foods being replaced; both less healthful and more healthful snacks were replaced. As expected Model 2, which did not replace whole fruits, non-starchy vegetables or whole grain snacks with tree nuts, resulted in better improvements and no significant declines in HEI-2010 component scores, with the exception of total dairy. However, because it is important to quantify both the positive and potentially negative impact of any model, we opted to use two models, one that universally replaced solid snack foods and another, which more judiciously replaced snack foods. Despite major improvements observed for many of the modeled dietary constituents evaluated, in many cases population mean intakes remained well below recommended levels (e.g., for sodium, fiber and potassium), suggesting that multiple other changes would need to be implemented simultaneously. For example, increasing whole grain, whole fruit or vegetable consumption, specifically through replacing refined grains and foods high in empty calories would also have tremendous population health impact. As such, these models should be viewed as an approach for quantifying and comparing the impact of various strategies, not as a prescription for specific changes an individual should make.

Substitution modeling complements and builds upon past studies comparing the diets of tree nut consumers and non-consumers. In past studies, based on 2005–2010 NHANES data, usual diets of tree nut consumers were also determined using two 24-h dietary recalls and the NCI Method [16]. That study assessed diet quality based on percentages of tree nut consumers below the estimated average requirement (EAR) or above the adequate intakes (AI) relative to non-consumers. Diets of tree nut consumers showed more favorable intakes for vitamins A and C, folate, calcium, iron, magnesium and zinc as well as potassium and fiber. The Healthy Eating Index 2005 score (HEI 2005) was higher for tree nut consumers as compared to non-consumers.

In cross-sectional studies, tree nut consumers and non-consumers were of necessity different people, who may differ by both observed (e.g., age) and unobserved or difficult to measure factors (e.g., health-seeking behavior). By contrast, substitution modeling offers some insight into how diets of individual consumers can be improved by making some small changes in food choices that are based, in part, on the existing eating patterns of the broader population as the tree nut composite was tailored based on population consumption patterns.

Past studies of tree nut consumers and non-consumers found that the two groups did not differ in their usual sodium intakes and both exceeded recommended adequate intake [16]. Given that tree nuts are generally very low in sodium, nut consumers, typical of the American diet consumed excessive amounts of sodium from other sources. In some past studies, tree nut consumers had lower sodium intakes than did non-consumers [15, 54]. Consistent with those data, the present modeling study showed that the sodium content in modeled diets was much lower than in observed diets. Given that a small increase was observed for potassium, there was a decline in the dietary sodium to potassium ratio, another index of diet quality. Comparable patterns were observed for almonds only modeling. These modeled data counter the perceptions that tree nut snacks may act as a vehicle for increasing dietary sodium.

The present study quantifies the impact of replacement of solid food snacks with tree nuts, suggesting that such a replacement may have a profound effect on population diet quality. However, the critical question moving forward is what policies and interventions may be most effective at increasing consumption of tree nuts at the population-level. Schools and childcare settings may be one environment where tree nut consumption could be increased. A number of tools were recently made available online by the US Department of Agriculture to help schools identify those food items that meet Smart Snacks Standards, which were implemented in 2014–15 [55]. Based on these Standards, almonds, dry roasted without salt in 1 oz packages (167 kcal) are compliant with the school guidelines, providing <200 kcal per serving [56], as nuts/seeds are exempt from the total fat threshold. Products consisting of only dried fruit with nuts and/or seeds with no added sugars or fats are also exempt from the saturated fat standard [57]. Additional policies that may promote nut consumption include the adoption of healthy vending policies, though the contribution of vending machines to snack intake at the population-level remains limited. Additional strategies to increase nut consumption may include social marketing and/or mass communication efforts to increase awareness regarding the health benefits of nuts or address potential misconceptions (e.g., that nuts consumption may lead to weight gain) [58]. In addition, targeted subsidies to reduce their cost in comparison to less healthful snacks might be an additional approach to increase their intake [59]. The efficacy of these approaches specific to increasing nut consumption has not yet been established.

The present study had a number of strengths. First, the data are nationally representative and we used the NCI method to estimate usual intake distributions for the entire population and by age group. Second, same-person replacement modeling complements past studies based on consumers and non-consumers of a specific food. Third, we used a composite tree nut, based on existing nut consumption patterns, which allows for a more precise assessment of the potential nutritional effects of behavioral changes. Further, the tree nut composite included both salted and unsalted nuts, weighted by consumption frequency. Therefore, the modeled results are representative of what would be observed if current consumption patterns continued.

Numerous limitations are also worth noting. First, all NHANES data were based on self-reports. Proxy reports were used for young children [60] and some foods may be under-reported, either through omission or error in recording portion sizes [61]. It is also possible that some participants reported tree nuts, while eating peanuts [15]. In addition, the results presented here are based on models and do not reflect actual behavior of individuals. Nonetheless, substitution modeling in the context of a specific meal or eating occasion, offers one way to test the potential nutritional impact of dietary guidance.

## Conclusion

Based on present modeling analyses, widespread replacement of solid food snacks with tree nuts or almonds would improve the quality of the diet. Even a partial replacement, exempting already nutrient rich snacks, had a significant positive effect on diet quality. Substitution modeling can provide added insight into the likely impact of following 2015–2020 DGAs on diet quality in the US.

## References

[1] Nicklas TA, O'Neil CE, Fulgoni VL (2014). Snacking patterns, diet quality, and cardiovascular risk factors in adults. BMC Public Health.

[2] Hess J, Slavin J (2014). Snacking for a cause: nutritional insufficiencies and excesses of U.S. children, a critical review of food consumption patterns and macronutrient and micronutrient intake of U.S. children. Nutrients.

[3] Piernas C, Popkin BM (2010). Trends in snacking among U.S. children. Health Aff (Millwood).

[4] Evans EW, Jacques PF, Dallal GE, Sacheck J, Must A (2015). The role of eating frequency on total energy intake and diet quality in a low-income, racially diverse sample of schoolchildren. Public Health Nutr.

[5] Hickson DA, Diez Roux AV, Smith AE, Tucker KL, Gore LD, Zhang L (2011). Associations of fast food restaurant availability with dietary intake and weight among African americans in the Jackson heart study, 2000–2004. Am J Public Health.

[6] Leung CW, Laraia BA, Kelly M, Nickleach D, Adler NE, Kushi LH (2011). The influence of neighborhood food stores on change in young girls' body mass index. Am J Prev Med.

[7] Choose MyPlate.gov. What are empty calories? Available at: http://www.choosemyplate.gov/weight-management-calories/calories/empty-calories.html. Accessed 16 May 2016.

[8] Huth PJ, Fulgoni VL, Keast DR, Park K, Auestad N (2013). Major food sources of calories, added sugars, and saturated fat and their contribution to essential nutrient intakes in the U.S. diet: data from the national health and nutrition examination survey (2003–2006). Nutr J.

[9] Whybrow S, Mayer C, Kirk TR, Mazlan N, Stubbs RJ (2007). Effects of two weeks’ mandatory snack consumption on energy intake and energy balance. Obesity.

[10] Rolls BJ, Roe LS, Kral TV, Meengs JS, Wall DE (2004). Increasing the portion size of a packaged snack increases energy intake in men and women. Appetite.

[11] Mazlan N, Horgan G, Whybrow S, Stubbs J (2006). Effects of increasing increments of fat- and sugar-rich snacks in the diet on energy and macronutrient intake in lean and overweight men. Br J Nutr.

[12] Zizza CA, Xu B (2012). Snacking is associated with overall diet quality among adults. J Acad Nutr Diet.

[13] Leidy HJ, Todd CB, Zino AZ, Immel JE, Mukherjea R, Shafer RS (2015). Consuming high-protein soy snacks affects appetite control, satiety, and diet quality in young people and influences select aspects of mood and cognition. J Nutr.

[14] U.S. Department of Health and Human Services and U.S. Department of Agriculture (2015). 2015 – 2020 Dietary guidelines for americans.

[15] O’Neil CE, Keast DR, Nicklas TA, Fulgoni VL (2012). Out-of-hand nut consumption is associated with improved nutrient intake and health risk markers in US children and adults: national health and nutrition examination survey 1999–2004. Nutr Res.

[16] O’Neil CE, Nicklas TA, Fulgoni VL (2015). Tree nut consumption is associated with better nutrient adequacy and diet quality in adults: national health and nutrition examination survey 2005–2010. Nutrients.

[17] Rehm CD, Penalvo JL, Afshin A, Mozaffarian D (2016). Dietary intake among US adults, 1999–2012. JAMA.

[18] Powell ES, Smith-Taillie LP, Popkin BM (2016). Added sugars intake across the distribution of US children and adult consumers: 1977–2012. J Acad Nutr Diet.

[19] Banfield EC, Liu Y, Davis JS, Chang S, Frazier-Wood AC (2016). Poor adherence to US dietary guidelines for children and adolescents in the national health and nutrition examination survey population. J Acad Nutr Diet.

[20] American Heart Association. Healthy Snacking. Available at: https://health.gov/dietaryguidelines/2015-scientific-report/pdfs/scientific-report-of-the-2015-dietary-guidelines-advisory-committee.pdf. Accessed 15 Nov 2016.

[21] American Diabetes Association. Snacks. http://www.diabetes.org/food-and-fitness/food/what-can-i-eat/food-tips/snacks.html? Accessed 15 Nov 2016.

[22] Academy of Nutrition and Dietetics. Smart Snacking for Adults and Teens. http://www.eatright.org/~/media/eatright%20files/nationalnutritionmonth/handoutsandtipsheets/nutritiontipsheets/smartsnackingforadultsandteens.ashx. Accessed 15 Nov 2016.

[23] O’Neil CE, Nicklas TA, III VLF (2016). Almond consumption is associated with better nutrient intake, nutrient adequacy, and diet quality in adults: national health and nutrition examination survey 2001–2010. Food Nutr Sciences.

[24] Bao Y, Han J, Hu FB, Giovannucci EL, Stampfer MJ, Willett WC (2013). Association of nut consumption with total and cause-specific mortality. N Engl J Med.

[25] Afshin A, Micha R, Khatibzadeh S, Mozaffarian D (2014). Consumption of nuts and legumes and risk of incident ischemic heart disease, stroke, and diabetes: a systematic review and meta-analysis. Am J Clin Nutr.

[26] Hu FB, Stampfer MJ, Manson JE, Rimm EB, Colditz GA, Rosner BA (1998). Frequent nut consumption and risk of coronary heart disease in women: prospective cohort study. BMJ.

[27] Mozaffarian D, Hao T, Rimm EB, Willett WC, Hu FB (2011). Changes in diet and lifestyle and long-term weight gain in women and men. N Engl J Med.

[28] Bes-Rastrollo M, Sabaté J, Gómez-Gracia E, Alonso A, Martínez JA, Martínez-González MA (2007). Nut consumption and weight gain in a Mediterranean cohort: The SUN study. Obesity (Silver Spring).

[29] Qualified health claims: letter of enforcement discretion—nuts and coronary heart disease. (Docket 02P-0505). pp. 1–4. US Food and Drug Administration. US Department of Health and Human Services; 2003. Available at: http://www.fda.gov/Food/IngredientsPackagingLabeling/LabelingNutrition/ucm072926.htm. Accessed on 16 May 2016.

[30] Monsivais P, Rehm CD (2012). Potential nutritional and economic effects of replacing juice with fruit in the diets of children in the United States. Arch Pediatr Adolesc Med.

[31] Rehm CD, Drewnowski A, Monsivais P (2015). Potential population-level nutritional impact of replacing whole and reduced-fat milk with low-fat and skim milk among US children aged 2–19 years. J Nutr Educ Behav.

[32] Salas-Salvado J, Casas-Agustench P, Salas-Huetos A (2011). Cultural and historical aspects of Mediterranean nuts with emphasis on their attributed healthy and nutritional properties. Nutr Metab Cardiovasc Dis.

[33] US Department of Health and Human Services: Centers for Disease Control and Prevention (CDC), National Center for Health Statistics. About the National Health and Nutrition Exmaination Survey (NHANES). Hyattsville; 2013.

[34] USDA Food and Nutrient Database for Dietary Studies 2011–2012. Food Surveys Research Group Home Page, http://www.ars.usda.gov/ba/bhnrc/fsrg U.S. Department of Agriculture, Agricultural Research Service, 2014.

[35] Food Patterns Equivalents Database. United States Department of Agriculture (USDA), Agricultural Research Service, 2014. Available at: http://www.ars.usda.gov/Services/docs.htm?docid=23871. Accessed 15 May 2016.

[36] U.S. Department of Agriculture. Scientific report of the 2015 Dietary Guidelines Advisory Committee 2015. 2015. https://health.gov/dietaryguidelines/2015-scientific-report/. Accessed 16 May 2016.

[37] Guenther PM, Casavale KO, Reedy J, Kirkpatrick SI, Hiza HA, Kuczynski KJ (2013). Update of the healthy eating index: HEI-2010. J Acad Nutr Diet.

[38] U.S. Department of Health and Human Services and U.S. Department of Agriculture (2010). 2010 Dietary guidelines for Americans.

[39] Tooze JA, Midthune D, Dodd KW, Freedman LS, Krebs-Smith SM, Subar AF (2006). A new statistical method for estimating the usual intake of episodically consumed foods with application to their distribution. J Am Diet Assoc.

[40] Tooze JA, Kipnis V, Buckman DW, Carroll RJ, Freedman LS, Guenther PM (2010). A mixed-effects model approach for estimating the distribution of usual intake of nutrients: The NCI method. Stat Med.

[41] Freedman LS, Guenther PM, Krebs-Smith SM, Kott PS (2008). A population’s mean Healthy Eating Index-2005 scores are best estimated by the score of the population ratio when one 24-h recall is available. J Nutr.

[42] HEI Tools for Researchers. Available at: http://epi.grants.cancer.gov/hei/tools.html. Accessed 15 May 2016.

[43] Guidance for Industry. A Food Labeling Guide (10. Appendix B: Additional Requirements for Nutrient Content Claims). US Department of Health and Human Services. January, 2013. Available at: http://www.fda.gov/Food/GuidanceRegulation/GuidanceDocumentsRegulatoryInformation/LabelingNutrition/ucm064916.htm. Accessed 15 May 2016.

[44] Food and Drug Administration. Guidance for Industry: A Food Labeling Guide (14. Appendix F: Calculate the Percent Daily Value for the Appropriate Nutrients). https://www.fda.gov/Food/GuidanceRegulation/GuidanceDocumentsRegulatoryInformation/LabelingNutrition/ucm064928.htm. Accessed 5 Nov 2016.

[45] National Center for Health Statistics. National Health and Nutrition Examination Survey. NCHS Research Ethics Review Board (ERB) Approval. Available at: http://www.cdc.gov/nchs/nhanes/irba98.htm. Accessed 15 May 2016.

[46] National Center for Health Statistics. National Health and Nutrition Examination Survey. Questionnaires, Datasets, and Related Documentation. https://www.cdc.gov/nchs/nhanes/nhanes_questionnaires.htm. Accessed 15 May 2016.

[47] National School Lunch Program and School Breakfast Program (2013). Nutrition standards for all foods sold in school as required by the healthy, hunger-free kids Act of 2010. Fed Regist.

[48] Piernas C, Popkin BM (2010). Snacking Increased among U.S. Adults between 1977 and 2006. J Nutrition.

[49] Sebastian RS, Wilkinson Enns C, Goldman JD. Snacking patterns of U.S. adults: What we eat in America, NHANES 2007–2008. Food Surveys Research Group Dietary Data Brief No. 4 June, 2011.

[50] Jacquier EF, Eldridge AL (2016). Eating dessert foods: behavioral patterns in American children. FASEB J.

[51] Gobbling Up Snacks: Cause or potential cure for childhood obesity? Available at: https://www.ers.usda.gov/amber-waves/2012/december/gobbling-up-snacks-cause-or-potential-cure-for-childhood-obesity/. Accessed 15 May 2016.

[52] Mattes RD, Dreher ML (2010). Nuts and healthy body weight maintenance mechanisms. Asia Pac J Clin Nutr.

[53] Hull S, Re R, Chambers L, Echaniz A, Wickham MS (2015). A mid-morning snack of almonds generates satiety and appropriate adjustment of subsequent food intake in healthy women. Eur J Nutr.

[54] O’Neil CE, Keast DR, Fulgoni VL, Nicklas TA (2010). Tree nut consumption improves nutrient intake and diet quality in US adults: an analysis of National Health and Nutrition Examination Survey (NHANES) 1999–2004. Asia Pac J Clin Nutr.

[55] Healthier school day. Tools for schools: Focusing on smart snacks. Available at: http://www.fns.usda.gov/healthierschoolday/tools-schools-focusing-smart-snacks. Accessed 15 May 2016.

[56] Alliance for a Healthier Generation. Alliance Product Calculator. Available at: https://www.healthiergeneration.org/take_action/schools/snacks_and_beverages/smart_snacks/alliance_product_calculator/. Accessed 16 May 2016.

[57] U.S. Department of Agriculture, Food and Nutrition Service. National School Lunch Program and School Breakfast Program: Nutrition Standards for All Foods Sold in School as Required by the Healthy, Hunger-Free Kids Act of 2010: Proposed Rules. Federal Register. 2013;78(27):50132–51. 23833807

[58] Pawlak R, Colby S, Herring J (2009). Beliefs, benefits, barriers, attitude, intake and knowledge about peanuts and tree nuts among WIC participants in eastern North Carolina. Nutr Res Pract.

[59] Afshin A, Penalvo J, Del Gobbo L, Kashaf M, Micha R, Morrish K, Pearson-Stuttard J, Rehm C, Shangguan S, Smith JD (2015). Mozaffarian D1 CVD prevention through policy: a review of mass media, food/menu labeling, taxation/subsidies, built environment, school procurement, worksite wellness, and marketing standards to improve diet. Curr Cardiol Rep.

[60] Troiano RP, Briefel RR, Carroll MD, Bialostosky K (2000). Energy and fat intakes of children and adolescents in the United States: data from the national health and nutrition examination surveys. Am J Clin Nutr.

[61] Lafay L, Mennen L, Basdevant A, Charles MA, Borys JM, Eschwège E, Romon M (2000). Does energy intake underreporting involve all kinds of food or only specific food items? Results from the Fleurbaix Laventie Ville Santé (FLVS) study. Int J Obes Relat Metab Disord.

